# Associations between waist circumference, metabolic risk and executive function in adolescents: A cross-sectional mediation analysis

**DOI:** 10.1371/journal.pone.0199281

**Published:** 2018-06-18

**Authors:** Anna Bugge, Sören Möller, Daniel R. Westfall, Jakob Tarp, Anne K. Gejl, Niels Wedderkopp, Charles H. Hillman

**Affiliations:** 1 Centre of Research in Childhood Health, Institute for Sport Science and Clinical Biomechanics, University of Southern Denmark, Odense, Denmark; 2 Department of Physiotherapy and Occupational Therapy, University College Copenhagen, Copenhagen, Denmark; 3 OPEN–Odense Patient Data Explorative Network, Odense University Hospital and Department of Clinical Research, University of Southern Denmark, Odense, Denmark; 4 Department of Psychology, Northeastern University, Boston MA, United States of America; 5 Sports Medicine clinic Orthopedic Department, Middelfart Hospital, Institute of Regional Health Research, University of Southern Denmark, Middelfart, Denmark; 6 Department of Physical Therapy, Movement, & Rehabilitation Sciences, Northeastern University, Boston MA, United States of America; Beijing Key Laboratory of Diabetes Prevention and Research, CHINA

## Abstract

The main objective of this study was to investigate the associations between waist circumference, metabolic risk factors, and executive function in adolescents. Methods: The study was cross-sectional and included 558 adolescents (mean age 14.2 years). Anthropometrics and systolic blood pressure (sysBP) were measured and fasting blood samples were analyzed for metabolic risk factors. A metabolic risk factor cluster score (MetS-cluster score) was computed from the sum of standardized sysBP, triglycerides (TG), inverse high-density lipid cholesterol (HDLc) and insulin resistance (homeostasis model assessment). Cognitive control was measured with a modified flanker task. Results: Regression analyses indicated that after controlling for demographic variables, HDLc exhibited a negative and TG a positive association with flanker reaction time (RT). Waist circumference did not demonstrate a statistically significant total association with the cognitive outcomes. In structural equation modeling, waist circumference displayed an indirect positive association with incongruent RT through a higher MetS-cluster score and through lower HDLc. The only statistically significant direct association between waist circumference and the cognitive outcomes was for incongruent RT in the model including HDLc as mediator. Conclusions: These findings are consonant with the previous literature reporting an adverse association between certain metabolic risk factors and cognitive control. Accordingly, these results suggest specificity between metabolic risk factors and cognitive control outcomes. Further, results of the present study, although cross-sectional, provide new evidence that specific metabolic risk factors may mediate an indirect association between adiposity and cognitive control in adolescents, even though a direct association between these variables was not observed. However, taking the cross-sectional study design into consideration, these results should be interpreted with caution and future longitudinal or experimental studies should verify the findings of this study.

## Introduction

Excess adiposity is one of the major public health issues concerning the western world today [1], and is considered among the most important factors in the etiology of metabolic dysfunctions and development of metabolic syndrome [2]. Waist-circumference, as a marker of abdominal fat mass, appears especially detrimental for metabolic regulation with a fourfold increase in type 2 diabetes risk when comparing high vs. low waist-circumference, even after controlling for body mass index (BMI) [3]. MetS and its individual risk factors are highly associated with the development of lifestyle diseases such as type 2 diabetes and cardiovascular disease [4, 5], and, more recently, research has suggested that both MetS and its individual components may also have deleterious consequences for brain health and cognition [6]. Increased metabolic risk has been associated with diseases such as dementia, Alzheimer’s disease, and general cognitive decline with age [7, 8].

Adiposity has also been associated with cognitive performance across the lifespan, and especially with executive functions [9, 10], which refers to a subset of goal-directed, self-regulatory operations encompassing the core processes of inhibition, working memory, and cognitive flexibility [11]. However, the biological pathways linking adiposity to cognitive performance are unclear. Various hypotheses have been suggested, and factors thought to mediate the association between adiposity and cognition include among others the risk factors included in the MetS (insulin resistance, dyslipidemia and hypertension), but also inflammation, vascular damage and subclinical atherosclerosis [12, 13]. One study in adults found that insulin sensitivity mediated the relationship between weight status measured by BMI and brain activation during working memory operations [14]. Given the importance of adipose tissue in the etiology of metabolic dysfunction [2] and the association between cognitive function and adiposity [9], it is conjectured that not only insulin resistance, but also other markers of metabolic dysfunctions such as dyslipidemia and high blood pressure, may be intermediate factors on the pathway between adiposity and cognitive function. However, to our knowledge, this potential mediation has not yet been investigated.

Some studies have found that overweight and obese children demonstrated poorer performance using several tasks that tap various aspects of executive function and other aspects of cognition compared to their normal-weight peers [15–17], while others have not observed associations between weight status and executive function [18]. Coinciding with the high prevalence of excess adiposity in youth, MetS is becoming more common in pediatric populations [19]. However, how the individual metabolic risk factors may affect executive function in youth is still unclear. To date, only a few studies have investigated this relationship [20–24]. These studies have found associations between metabolic risk and certain measures of executive function, indicating that cognitive function could be compromised by metabolic dysregulation even in the first decades of life. These findings further suggest the possibility that MetS may have differential effects on the various subdomains of executive function. In addition, diverse results regarding which metabolic risk factors are associated with executive function have been found [20–24]. Moreover, some researchers have questioned the use of pediatric MetS definitions in healthy youth populations for several reasons [25, 26]. These challenges have led to the development of a method to identify children and adolescents not yet having MetS, but with a higher cardiovascular risk than their peers; the continuous metabolic risk factor cluster score (MetS-cluster score) [26–28]. Clustering of metabolic risk factors have been identified in children as young as nine years old [26]. To our knowledge, no studies have investigated whether the metabolic risk factors included in MetS including a MetS-cluster score mediate the association between adiposity and cognition in youth.

Therefore, the aim of this investigation was to explore the association between waist circumference, metabolic risk factors including a MetS-cluster score, and executive function. A secondary aim was to assess whether the association between waist-circumference and executive function is mediated by the effect of excess adipose tissue on the metabolic risk factors. Based on the current literature, our hypothesis is that both a higher waist circumference and higher levels of the metabolic risk factors are associated with poorer executive function in adolescents, and that the association between adiposity and executive function is partly mediated by higher levels of the individual and combined metabolic risk factors.

## Materials and methods

### Study design and participants

This investigation is part of the Childhood Health, Activity, and Motor Performance School Study Denmark (CHAMPS study-DK), a large-scale, quasi-experimental study based on a natural experiment. At baseline in 2008, all 19 schools in the municipality of Svendborg, Denmark, were invited to participate in the study and ten schools agreed. The study and the methods used have been described in detail elsewhere [29], and only measurements pertinent to this paper are included below. Data for the present investigation are solely from the most recent follow-up conducted in 2015 and this investigation is therefore cross-sectional. For this follow-up parents/legal guardians of 745 adolescents (from a total of 1457 approached) provided written informed consent, and 705 adolescents participated in the testing. Of these, 558 had complete data and were included in the present analysis. The CHAMPS study–DK was approved by the Regional Committees on Health Research Ethics for Southern Denmark, Region of Southern Denmark (Project number: S-20080047 and S-20140105).

### Measurements

All blood samples, anthropometric, and physiological measurements were collected on one day and cognitive tests were collected on a separate day.

#### Anthropometrics

Body mass was measured to the nearest 0.1 kg on an electronic scale (Tanita BWB-800S, Tanita Corporation, Tokyo, Japan) with participants wearing shorts and t-shirts. Stature was measured to the nearest 0.5 cm using a portable stadiometer (SECA 214, Seca Corporation, Hamburg, Germany). BMI was calculated as weight (kg)/height (m) ^2^, and normal weight, overweight and obesity categories were calculated as suggested by Cole and colleagues [30]. Waist circumference (WC) was measured at the level of the umbilicus after a light expiration. Two measurements were performed and if the results differed by more than one cm a third measurement was performed and the mean of the two nearest measurement results were used. Pubertal status was self-assessed using the Tanner pubertal stages questionnaires [31]. Privately, participants were asked to assess which category they belonged to by looking at pictures of five pubertal stages (breast development for girls and pubic hair for boys).

#### Blood samples

Blood samples were collected between 8:00–10:00 am after an overnight fast (min 8 hours). Blood samples were kept on ice, handled in the laboratory within four hours and subsequently stored at -80°C until analyzed. Triglycerides (TG), glucose and high-density lipid cholesterol (HDLc) were analyzed by quantitative determination using enzymatic, colorimetric method on a Roche/Hitachi cobas c system (Roche, Mannheim, Germany). Insulin was analyzed using solid phase enzyme labelled chemiluminescent immunometric assay (Beckman Coulter GmbH, Vienna, Austria). Insulin resistance was estimated by the homeostasis model assessment (HOMA-IR); glucose (mmol/L) x insulin (μU/mL)/22.5 [32].

#### Blood pressure

Blood pressure was measured after five minutes of seated rest using an automated oscillometric blood pressure monitor (Omron 705IT, Omron, Kyoto, Japan). The measurement was repeated a minimum of five times at two-minute intervals until stable. Mean of the three last recordings of systolic blood pressure (sysBP) was used in the analyses.

#### The composite risk factor score

The composite risk factor score was comprised of the mean of standardized residuals (z-scores, standardized by age, gender and pubertal status) of HDLc, TG, sysBP and HOMA-IR. Before generating the MetS-cluster score non-normal distributed variables were natural log-transformed (HOMA-IR and TG). HDLc was multiplied by -1, as a higher value of this variable is desirable for metabolic health. SysBP was further standardized by height. Furthermore, the blood markers were standardized by weekday as preliminary analyses of the data revealed an association between weekday and TG and insulin, a phenomenon resembling previous investigations [33]. A higher value of the composite risk factor score represents a less favorable risk profile. For analyses the MetS-cluster score was standardized to mean = 0.0 and standard deviation (SD) = 1.0 for direct comparison to the other variables.

#### Executive function task

To assess inhibition all participants performed a modified flanker task [34]. Five arrows were presented on a screen and participants were instructed to respond as quickly and accurately as possible to the directionality of the central target arrow amid an array of flanking arrows. A target arrow pointing to the right “>” required a right-handed response and a target arrow pointing to the left “<” required a left-handed response. Flanking arrows were either congruent (>>>>> or <<<<<) or incongruent (>><>> or <<><<) relative to the target arrow, and the two conditions is thought to require variable amounts of interference control. Participants completed a practice block of 20 trials before the task was performed. The test consisted of two blocks of 75 trials with congruent and incongruent trials being presented randomly and with equal probability. Stimuli were presented for 120 milliseconds (ms) with a response window between 200–1470 ms after the onset of the stimulus. A randomized inter-stimulus interval of 1250, 1350, 1450 or 1550 ms separated each trial and a 30 second break separated the two blocks. Response accuracy (ACC) and reaction time (RT) were assessed for congruent and incongruent trials separately. Interference scores were calculated as the difference in ACC and RT between congruent and incongruent conditions. Participants having an overall ACC of <50% or a RT above > 3 SD were discarded from further analysis.

#### Socioeconomic status

The female guardian’s highest completed education was obtained from a questionnaire and used as an indicator of socioeconomic status (SES) [35]. Information on male guardian was used, when data was not available for the female guardian. Categories included completion of: 1) 10th grade or less, 2) vocational education, 3) high school education, 4) short tertiary education, 5) bachelor’s degree or equivalent, 6) master’s degree or higher.

#### Statistical analyses

Differences between boys and girls and between participants included and excluded in the main analyses were analyzed using unpaired t-tests for continuous and chi-squared test tests for categorical variables.

**Fig 1:**
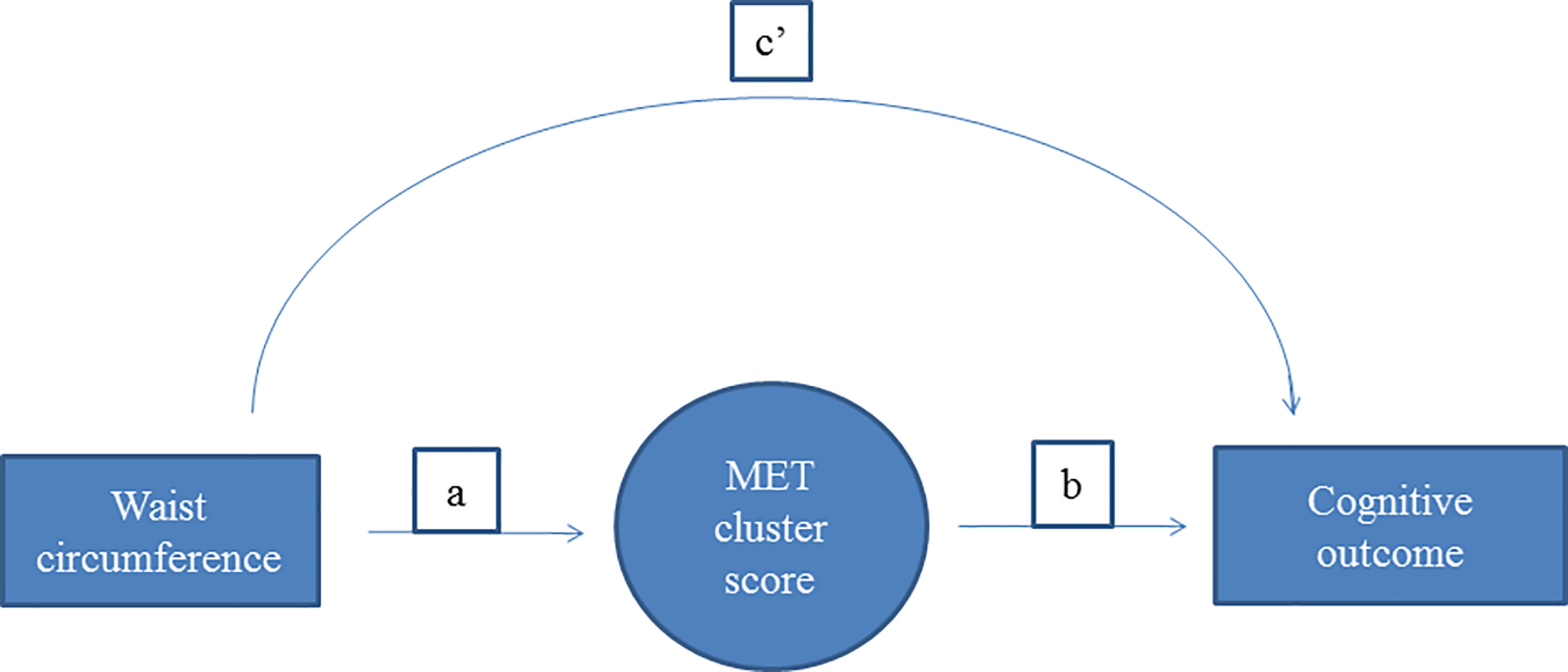
Schematic illustration of the path diagrams of the structural equation model. Schematic illustration of the path diagrams of the structural equation model; waist circumference →cognition, with MET cluster score as the mediating factor. Directs associations = c’, indirect associations = ab, total associations = c’ + ab. All associations adjusted for sex, pubertal status, parental education and age. MET cluster score: sum of z-scores of HOMA, HDL cholesterol (inverse), systolic blood pressure and triglyceride.

The associations between waist circumference (standardized by sex and age), metabolic risk factors (standardized by sex and age) and cognitive performance outcomes were assessed using linear regression models adjusting for potential confounders (sex, pubertal status, SES, and age). Structural equation modeling (SEM) was used to further explore the hypothesized link between waist-circumference and cognition by assessing potential mediation by the metabolic risk factors. Separate models including each of the individual metabolic risk factors and the MetS-cluster score as mediating factors in the association between waist circumference and cognitive performance adjusting for the potential confounders were estimated. Fig 1 shows the hypothesized path model with the direct and indirect associations between waist circumference and cognitive outcomes through the metabolic risk factors.

Direct (c’ in Fig 1) and indirect (through the risk factors, ab in Fig 1) associations between the standardized waist circumference and the (unstandardized) cognitive performance outcomes were calculated by maximum likelihood estimation of the SEM model with Satorra-Bentler standard error estimates. To evaluate the goodness-of-fit of the SEM models we evaluated the coefficient of determination of the model, as well as the Bentler-Raykov squared multiple correlation coefficient for each equation in the models. Analyses were re-run including flanker task RT outliers to investigate the effect of this exclusion criterion. Residuals were checked for normality across all analyses, and variables that did not result in normally distributed residuals were transformed by the natural logarithm. All analyses were carried out in Stata/SE 14.1.

## Results

There were no differences in age, height or weight between participants included in this investigation (n = 558) and those excluded from these analyses because of missing data (n = 147) (all p’s ≥ 0.05). However, BMI differed between the groups (p = 0.04), such that the subjects excluded from these analyses had a slightly higher BMI (mean 19.98, SD 2.85) compared to the included participants (mean 19.45, SD 2.66). Sex-specific distributions and differences of included variables are presented in Table 1.

**Table 1:** Characteristics of participants by sex (values are mean and SD unless otherwise stated).

	Boys N = 314	Girls N = 347	P-values for differences between sexes
**Age (years)**	14.39 (1.28)	13.97 (1.27)	≤ 0.0001
**Body weight (kg)**	55.21 (10.39)	53.77 (11.33)	0.12
**Height (cm)**	167.14 (9.43)	166.27 (9.81)	0.29
BMI*	19.53 (2.86)	19.38 (2.57)	0.09
**Normal weight/overweight/obese (percent)**^**a**^	91.7% / 7.1% / 1.2%	89.8% / 8.8% / 1.4%	0.668
Waist circumference (cm)*	72.49 (7.65)	71.11 (7.83)	0.03
HOMA-IR*	2.03 (1.32)	2.06 (1.56)	0.90
**Systolic BP (mmHg)**	107.99 (8.66)	107.07 (8.59)	0.21
Triglycerides (mmol/L)*	0.77 (0.30)	0.76 (0.37)	0.53
**HDL-cholesterol (mmol/L)**	1.42 (0.34)	1.48 (0.37)	0.08
METs cluster score*	0.02 (0.46)	-0.04 (0.51)	0.12
**Parental education**^**b**^ **(percent reporting in each category)**			0.94
High school education or less	8.7	7.2	
Vocational education	29.6	29.9	
Short tertiary education	9.9	13.3	
Bachelor or equivalent	44.7	42.8	
Master degree or higher education	7.1	6.8	
**Tanner stages**^**c**^			
1–2, 3, 4, 5 (percent)	5.5/33.7/48.7/12.1	9.8/34.0/43.5/12.6	0.23
**Flanker task**			
Reaction Time (ms)			
Congruent	446.1 (55.5)	453.6 (55.7)	0.11
Incongruent	537.0 (76.8)	550.0 (74.9)	0.04
Response Accuracy (%)			
Congruent*	95.4 (7.4)	95.8 (5.7)	0.37
Incongruent*	81.0 (14.0)	82.2 (12.7)	0.30
Interference score			
Reaction time (ms)	90.9 (43.1)	96.4 (40.4)	0.12
Accuracy (%)	14.3 (10.7)	13.7 (10.2	0.45

^a^ BMI categories according to International Obesity Task Force age- and sex specific cutoff points [30].

^b^ Maternal or female guardians highest completed education was used as the parental education indicator if available.

^c^ Category 1 and 2 are collapsed, as there were few participants in these categories.

*Not normally distributed data, transformed using the natural logarithm for analyses.

Boys were older, and had higher BMI and waist circumference compared to girls, and girls had higher HDLc and longer incongruent RT compared to boys (all p’s < 0.05).

Table 2 shows associations between waist circumference (not controlled for the putative mediators), metabolic risk factors and the modified flanker task outcomes. HDLc was negatively associated with congruent and incongruent RT after adjusting for confounders (β = -5.15 and -6.93, p = 0.030 and 0.031, respectively), indicating that participants with lower HDLc had longer RT. Furthermore, standardized TG level was positively associated with incongruent RT (β = 7.15, p = 0.024) and the RT interference score (β = 3.63, p = 0.039) after adjusting for confounders. No other significant associations were observed.

**Table 2:** Associations (total associations) between waist circumference, metabolic risk factors and the flanker task (β-values and 95% CI).

	Reaction Time		Accuracy		Interference score	
	Congruent	Incongruent	Congruent	Incongruent	Reaction time	Accuracy
**Z-Waist circumference**	2.65 (-2.02–7.32)	3.02 (-3.33–9.37)	-0.35 (-0.90–0.21)	-1.03 (-2.16–0.10)	0.37 (-3.14–3.89)	0.68 (-0.21–1.57)
**Z-HOMA-IR**	2.88 (-1.76–7.52)	3.09 (-3.22–9.40)	-0.38 (-0.93–0.18)	-0.59 (-1.72–0.54)	0.21 (-3.29–3.71)	0.21 (-0.67–1.10)
**Z-HDL-cholesterol**	-5.15 (-9.78 – -0.51)*	-6.93 (-13.23 – -0.63)*	-0.09 (-0.64–0.47)	-0.50 (-1.63–0.63)	-1.78 (-5.28–1.72)	0.42 (-0.47–1.30)
**Z-Systolic BP**	0.35 (-4.33–5.04)	-0.61 (-6.98–5.76)	-0.43 (-0.99–0.13)	-0.33 (-1.47–0.81)	-0.96 (-4.48–2.56)	-0.10 (-0.99–0.79)
**Z-Triglycerides**	3.52 (-1.07–8.10)	7.15 (0.93–13.36)*	-0.22 (-0.77–0.32)	-0.26 (-1.37–0.86)	3.63 (0.19–7.07)*	0.03 (-0.84–0.91)
**Z_METs cluster score** **(z-HOMA+z-HDL(inv)+** **z-SysBP+z-TG)**	4.47 (-0.16–9.10)	6.06 (-0.23–12.34)	-0.35 (-0.90–0.21)	-0.18 (-1.31–0.94)	1.59 (-1.90–5.08)	-0.17 (-1.05–0.72)

Linear regression models adjusted for sex, age, puberty and parental education.

* P<0.05. β-values are partially standardized and should be interpreted as the changes in absolute values of the cognitive outcomes for each standard deviation change in the exposure (metabolic risk factors). Not adjusted for potential mediators.

Results from the SEMs used to investigate possible mediations are presented in Table 3.

**Table 3:** SEM analyses of associations between the flanker task and waist circumference through the metabolic risk factors (β-values and 95% CI).

		Reaction Time		Accuracy		Interference score	
		Congruent	Incongruent	Congruent	Incongruent	Reaction time	Accuracy
**Waist circumference–cognition:** **including MET cluster score as mediator**	***Direct associations*** **Waist circumference (c’)**	1.26 (-3.63–6.14)	1.07 (-5.57–7.70)	-0.26 (-0.84–0.33)	-1.09 (-2.28–0.10)	-0.19 (-3.88–3.49)	0.83 (-0.10–1.76)
	***Indirect associations*** **Waist circumference → MET cluster score → (a**^**1**^ **b**^**1**^**)**	1.39 (-0.31–3.08)	1.95 (0.35–4.26)*	-0.09 (-0.29–0.11)	0.06 (-0.34–0.47)	0.57 (-0.69–1.83)	-0.15 (-0.47–0.16)
**Waist circumference–cognition:** **including HDLc as mediator**	***Direct associations*** **Waist circumference (c’)**	1.82 (-2.84–6.47)	1.89 (-4.44–8.21)	-0.37 (-0.93–0.18)	-1.15 (-2.28 –-0.02)*	0.07 (-3.45–3.59)	-0.77 (-0.11–1.66)
	***Indirect associations*** **Waist circumference → HDLc → (a**^**1**^ **b**^**1**^**)**	0.82 (-0.06–1.72)	1.13 (-0.08–2.34)*	0.03 (-0.07–0.12)	0.12 (-0.08–0.32)	0.30 (-0.31–0.92)	-0.09 (-0.25–0.06)
**Waist circumference–cognition:** **including HOMA score as mediator**	***Direct associations*** **Waist circumference (c’)**	2.09 (-2.64–6.82)	2.43 (-3.99–8.86)	-0.27 (-0.84–0.02)	-0.94 (-2.09–0.21)	0.34 (3.22–3.91)	0.67 (-0.23–1.56)
	***Indirect associations*** **Waist circumference → HOMA score → (a**^**1**^ **b**^**1**^**)**	0.56 (-0.55–1.66)	0.59 (-0.91–2.08)	-0.07 (-0.21–0.06)	-0.09 (-0.35–0.18)	0.03 (-0.79–0.85)	0.01 (-0.19–0.22)
**Waist circumference–cognition:** **including Sys BP as mediator**	***Direct associations*** **Waist circumference (c’)**	2.76 (-2.03–7.54)	3.44 (-3.06–9.95)	-0.25 (-0.82–0.32)	-1.01 (-2.17–0.15)	0.69 (-2.92–4.29)	0.76 (-0.14–1.67)
	***Indirect associations*** **Waist circumference → Sys BP → (a**^**1**^ **b**^**1**^**)**	-0.11 (-1.42–1.20)	-0.42 (-2.21–1.36)	-0.10 (-0.26–0.06)	0.01 (-0.33–0.30)	-0.31 (-1.30–0.68)	-0.08 (-0.33–0.16)
**Waist circumference–cognition:** **including TG as mediator**	***Direct associations* Waist circumference (c’)**	2.15 (-2.50–6.81)	1.97 (-4.34–8.27)	-0.32 (-0.87–0.24)	-1.01 (-2.14–0.12)	-0.19 (-3.68–3.31)	0.69 (-0193–1.57)
	***Indirect associations* Waist circumference → TG → (a**^**1**^**b**^**1**^**)**	0.49 (-0.26–1.24)	1.05 (-0.06–2.16)	-0.03 (-0.11–0.06)	0.02 (-0.19–0.15)	0.56 (-0.05–1.17)	-0.01 (-0.14–0.12)

Structural equation models (SEM).

* p<0.05. β-values are partially standardized and should be interpreted as the changes in absolute values of the cognitive outcomes for each standard deviation change in the exposure (metabolic risk factors).

When analyzed in separate models for each putative mediator, waist circumference had no direct association with any of the cognitive outcomes (p’s > 0.05), except in the model with HDLc as a mediator where a direct negative association was found between waist circumference and incongruent RT (estimate -1.15, p = 0.047). Waist circumference displayed an indirect positive association with incongruent RT through a higher MetS-cluster score (estimate of indirect association 1.95, p = 0.049). This suggests that for a one standard deviation increase in waist circumference, incongruent RT is 1.95 milliseconds longer owing to the association between waist circumference and the metabolic risk score. Furthermore, waist circumference was found to have a significant indirect positive association with incongruent RT through HDLc (estimate of indirect association 1.13, p = 0.046). Waist circumference was not significantly associated with the cognitive outcomes through any of the other possible mediators (p’s > 0.05). The models resulted in overall Bentler-Raykov squared multiple correlation coefficients between 0.07 and 0.19 indicating an important contribution of waist circumference and metabolic risk factors on outcomes while, as expected, still leaving a large part of the variation in executive function unexplained. All model assumptions were found to be met and the sensitivity analyses including RT outliers did not change the main conclusions.

## Discussion

In line with the hypothesis of the present study, the main findings were that HDLc and TG were associated with inhibitory control. Specifically, a lower level of HDLc was associated with generally poorer performance as indicated by longer RTs in the flanker task in both congruent and incongruent trials. Likewise, higher levels of TG related to selectively poorer performance as indicated by longer incongruent RT and greater RT interference. In contrary to our proposed hypothesis, no associations were found between flanker task performance and waist circumference, the MetS-cluster score, insulin resistance or sysBP, indicating specificity in the link between cognitive control and the metabolic risk factors.

### Executive function and metabolic risk factors

Previous studies have indicated heterogeneous results regarding which individual metabolic risk factors are associated with executive functions. Similar to the present results, Scudder et al. (2015) found that unfavorable levels of HDLc and TG were associated with longer flanker task RT. However, after adjusting for putative confounders, the association with TG did not remain significant [20]. On the contrary, data from the NHANES study showed no association between cognitive performance measured with the digit span task (a measure of working memory) and exceeding the MetS cut point or being in the highest or lowest quartiles for HDLc or TG, respectively [22, 24]. Likewise, studies in adults are also conflicting (for review see e.g. [12]). However, a meta-analysis showed a consistent association between total cholesterol in midlife and future risk for cognitive impairment and dementia [36]. Interestingly, a similar relationship was not observed in older age-groups. Exploring the underlying mechanisms, different studies have found that dyslipidemia contributes to microvascular disease and vascular damage in the brain [12, 37]. However, the exact mechanisms by which dyslipidemia influence cognitive functioning are still unclear, mainly because dyslipidemia rarely occurs in isolation, rather is usually accompanied by hypertension and impaired glucose metabolism [37]. Future studies are needed both to address possible differences in the association between dyslipidemia and cognitive impairment across the lifespan, and the biological mechanisms driving this association.

In the present study, no association between the flanker task variables and insulin resistance measured by HOMA-IR was found. Previous studies in youth have used fasting glucose as a measure of glucose metabolism, and have likewise not observed any associations with executive functions [20, 22, 24]. In adults, an association between diabetes and cognitive impairment (including dementia) has been well established (e.g. [38, 39]). Insulin resistance has been proposed as a key factor linking the association between MetS and cognitive impairment [40], and hyperglycemia has been found to be an important contributor of the association between MetS and cognition [41]. The discrepancy between studies in youth and adults suggests that dysregulation of glucose metabolism in the early stages might not affect cognition, whereas more severe, later stage insulin resistance and concomitant severe hyperglycemia might. The mechanisms by which type 2 diabetes and insulin resistance affect cognition and brain function includes disrupted white matter integrity, vascular micro- and macro-abnormalities in the brain and brain atrophy (for review see e.g. [12, 13]). These conditions are only seen in late stages type 2 diabetes and after prolonged hyperglycemia [37], and not typically observed in youth populations. This might explain the discrepancies in results from different age groups.

The present study does not find any associations between sysBP and executive functions, which corroborates several studies in youth [20, 23]. However, others found that children and adolescents with sysBP ≥ 90th percentile performed poorer on a working memory task compared to their normotensive peers [22, 42]. As such, it has been suggested that hypertension may cause impaired cognitive function via impairment of vascular reserve and microvascular disease [43], which are consequences of severe and prolonged hypertension and therefore, to our knowledge, not found in healthy youth.

Taken together, there is no agreement between studies on which of the metabolic risk factors are associated with executive functions, and it is possible that individual differences in health status, adiposity, fitness level etc. across study populations and the different age groups included contribute to the observed heterogeneity. Despite the lack of consistency among studies, current evidence indicates that optimal cognitive function may be compromised by metabolic dysregulation even in the first decades of life.

Results from our study did not show any associations between the MetS-cluster score and measures of executive function. To date, only a few studies have investigated the associations between clustered metabolic risk and executive functions in children or adolescents [20–23]. Yau et al. (2012) found that adolescents with MetS performed worse on measures of mental flexibility compared to adolescents without symptoms of MetS. However, none of the other tests of executive function were associated with MetS status [21]. These findings were combined with reduced structural integrity in the brains of adolescents with MetS compared to their healthy counterparts [21]. Similarly, Rubens et al. (2016) found that adolescents with MetS had lower working memory and attentional performance as well as lower reading scores, whereas no differences were observed for perceptional and visuospatial reasoning skills using the Wechsler Intelligence Scale for Children Revised. Additionally, Scudder and colleagues (2015) performed a study in children (mean age 7.5 years) and found that those not meeting any MetS risk-factor criteria had shorter RTs on a modified flanker task and performed better during the more difficult conditions of the task compared to children presenting with one or more risk-factors for MetS. Accordingly, current evidence suggests an association between MetS and some measures of executive function, but highlights the possibility that MetS may have differential effects on the various executive function subdomains.

### Possible mechanisms driving the association between adiposity and executive functions

Most studies, both in youth and adults, agree on a negative relationship between weight status and cognitive control [10, 15–17]. Many possible mechanisms linking adiposity and cognition have been proposed including all the metabolic risk factors composing MetS, but also factors like vascular damage, low grade inflammation and hyper glucocorticoids have been proposed as possible contributing candidates [13]. In the present study waist circumference displayed an indirect positive association with incongruent RT through a higher MetS-cluster score and an indirect positive association with both congruent and incongruent RT through lower HDLc. This suggests the negative impact of excess adiposity on cognition seen in some studies (e.g. [15–17]) may be mediated, at least in part, by the effect of adipose tissue on these metabolic risk factors. However, in mediation analyses performed on cross-sectional data controlling for prior level of the mediators or the outcome (to reduce potential reverse causality bias) is not possible, which renders it impossible to conclude anything on causality [44]. Therefore, future studies should verify this relationship, optimally in a prospective study design and including a larger array of adiposity-related metabolic markers. One previous cross-sectional study in adults found that insulin sensitivity mediated the relationship between weight status, measured by BMI, and brain activation during a working memory task [14], which is in contrast to our findings on insulin resistance. These discrepancies could be due to different measurements of exposure, mediator and outcome, and also to the differences in age groups studied. Our findings of no direct association between adiposity and cognitive control is raising the intriguing question that it might not be excessive adiposity *per se*, but the concomitant metabolic disturbances in traditional metabolic risk factors that drive the association with impaired cognition. It has been shown that these biological markers can be improved by lifestyle changes even without substantial weight loss [45, 46], which is encouraging given the knowledge that weight loss can be difficult to maintain.

### Limitations

A clear limitation in the present study is the cross-sectional study design making it impossible to conclude causality. Also, it is possible that lower executive functions could predispose weight-gain associated with impulsive behavior such as e.g. snacking or low physical activity levels (i.e. reverse causality). Further, residual or unmeasured confounding cannot be rejected. Inclusion of a more comprehensive cognitive test battery could have extended our results to other aspects of executive function, memory and cognition in general, allowing for an understanding of the general versus selective nature of the relationship of MetS on cognition. Also, it is possible that other biologic consequences of excess adiposity, not measured in this study, could mediate the association between adiposity and executive control, and future studies could include e.g. measures of vascular damage or subclinical atherosclerosis. Finally, the sampled population in this study is, according to the biological characteristics, a metabolic healthy and relatively normal weight cohort of young Danish people, and might not be representative for adolescents in an international context. The variation in waist-circumference for age is therefore likely to be smaller than observed elsewhere, potentially explaining the absence of association between waist circumference and cognitive outcomes.

## Conclusion

In summary, results from the present study demonstrated an association between HDLc, TG and executive function, while no association was observed for waist circumference. However, waist circumference was negatively and indirectly associated with executive function, as mediated through HDLc and the MetS-cluster score. Our findings and results from other studies suggest that optimal cognitive function could be compromised by metabolic dysregulation already in adolescence. These results highlight the necessity of developing future comprehensive health initiatives that simultaneously can contribute to healthy weight, overall metabolic health and executive function, leading to better academic performance and general well-being in young people.
